# Fault Detection in Power Distribution Networks Based on Comprehensive-YOLOv5

**DOI:** 10.3390/s23146410

**Published:** 2023-07-14

**Authors:** Shengsuo Niu, Xiaosen Zhou, Dasen Zhou, Zhiyao Yang, Haiping Liang, Haifeng Su

**Affiliations:** Hebei Provincial Key Laboratory of Power Transmission Equipment Security Defense, North China Electric Power University, Baoding 071003, China

**Keywords:** deep learning, Comprehensive-YOLOv5, real-time fault detection, power distribution networks, lightweight

## Abstract

Real-time fault detection in power distribution networks has become a popular issue in current power systems. However, the low power and computational capabilities of edge devices often fail to meet the requirements of real-time detection. To overcome these challenges, this paper proposes a lightweight algorithm, named Comprehensive-YOLOv5, for identifying defects in distribution networks. The proposed method focuses on achieving rapid localization and accurate identification of three common defects: insulator without loop, cable detachment from the insulator, and cable detachment from the spacer. Based on the You Only Look Once version 5 (YOLOv5) algorithm, this paper adopts GhostNet to reconstruct the original backbone of YOLOv5; introduces Bidirectional Feature Pyramid Network (BiFPN) structure to replace Path Aggregation Network (PANet) for feature fusion, which enhances the feature fusion ability; and replaces Generalized Intersection over Union GIOU with Focal Extended Intersection over Union (Focal-EIOU) to optimize the loss function, which improves the mean average precision and speed of the algorithm. The effectiveness of the improved Comprehensive-YOLOv5 algorithm is verified through a “morphological experiment”, while an “algorithm comparison experiment” confirms its superiority over other algorithms. Compared with the original YOLOv5, the Comprehensive-YOLOv5 algorithm improves mean average precision (mAP) from 88.3% to 90.1% and increases Frames per second (FPS) from 20 to 52 frames. This improvement significantly reduces false positives and false negatives in defect detection. Consequently, the proposed algorithm enhances detection speed and improves inspection efficiency, providing a viable solution for real-time detection and deployment at the edge of power distribution networks.

## 1. Introduction

The rapid growth of power components and transmission lines has increased the difficulty of power supply and the workload of maintenance and repair of the grid system [1,2,3]. Eighty to ninety percent of power system faults occur in the distribution network. Distribution lines are susceptible to factors such as lightning, storms, and magnetic fields during operation. Once a fault occurs, it will have a significant impact on the economy, industrial production, and the normal lives of residents [4,5,6].

In recent years, with the advancement of unmanned aerial vehicle (UAV) technology, UAV aerial photography has been increasingly applied in power distribution line inspections. By analyzing aerial images, defects can be promptly identified and repaired, ensuring the normal operation of distribution lines. References [7,8] have investigated the feasibility of intelligent UAV inspections by analyzing the required image data and autonomous navigation techniques. Reference [9] improved the deep convolutional neural network (DCNN) by designing an adaptive strategy for the localization of the DCNN model, thereby extending the endurance of UAV inspections. Reference [10] employed a cascaded Faster R-CNN network model for insulator detection and self-destruction recognition on UAV images. It utilized feature pyramids to extract target features and combined region proposal networks to generate candidate regions, resulting in improved accuracy for insulator self-destruction detection and recognition. These studies have effectively enhanced the accuracy of fault recognition in power distribution lines by increasing the depth and complexity of the models. However, deploying large and bulky models on mobile devices not only requires high-performance hardware support but also significantly reduces the speed of fault recognition in distribution lines.

Deep convolutional neural networks have demonstrated significant superiority in the field of fault localization and recognition in power distribution lines. Current research can be primarily divided into two categories. The first category includes two-stage object detection models represented by the Region-based Convolutional Network method (R-CNN) [11], Fast Region-based Convolutional Network method (Fast R-CNN) [12], and Faster Region-based Convolutional Network method (Faster R-CNN) [13]. Reference [14] proposed a parallel convolutional neural network (P-CNN) training method based on transfer learning, which constructed a dual-branch convolutional neural network with fault classification and localization branches. Reference [15] employed a genetic algorithm to optimize the parameters of the support vector machine model, creating a multi-support vector machine model diagnostic approach for electrical quantity fault information features. However, two-stage object detection algorithms have a large number of network parameters, requiring more resources and a longer detection time. Another category of representative algorithms includes the YOLO (You Only Look Once) series [16,17,18,19,20], which includes YOLOv1, YOLOv2, YOLOv3, YOLOv4, and YOLOv5, as well as the Single Shot MultiBox Detector (SSD) algorithm [21]. This paper focuses on the YOLOv5 algorithm, which not only maintains high accuracy but also features fast detection speed, short processing time, and low memory usage. This study addresses the problem of localization and identification of defects in power distribution components and proposes an improved version of the Comprehensive-YOLOv5 lightweight neural network model for end-to-end defect localization and identification. Based on the original YOLOv5, the main body and neck are reconstructed using GhostNet [22] to reduce the model size and improve computational speed. The Bi-directional Feature Pyramid Network (BiFPN) [23,24,25,26,27] structure is employed to replace the Path Aggregation Network (PANet) [28,29,30,31,32,33] for feature fusion, enhancing computational accuracy. The Focal Extended Intersection over Union (Focal-EIOU) loss function is used instead of Generalized Intersection over Union (GIOU) for optimization [34,35]. This approach ultimately achieves fast and accurate recognition and detection of three typical defects in power distribution networks: insulator without loop, cable detachment from insulator, and cable detachment from spacer.

## 2. Distribution Grid Fault Detection Network

### 2.1. Network Model of YOLOv5

**Figure 1:**
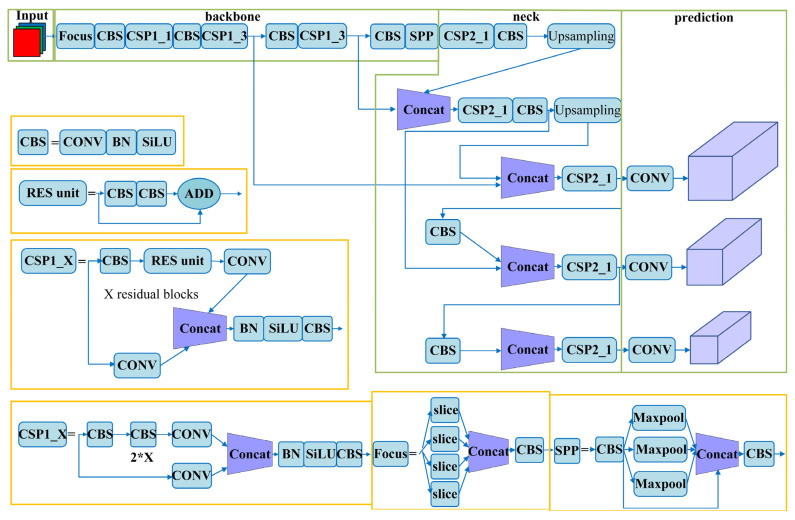
Network Model of YOLOv5.

The background of power distribution networks is complex and diverse, and different angles and lighting conditions can potentially interfere with unmanned aerial vehicle (UAV) fault detection. Therefore, it is crucial to select a robust and highly resilient network model. Compared to region-based two-stage object detection models such as R-CNN, Fast R-CNN, and Faster R-CNN, the YOLOv5 algorithm belongs to a one-stage object detection model. It directly predicts the relative positions of candidate bounding boxes and achieves object classification and bounding box prediction. The network structure of YOLOv5 is illustrated in Figure 1.

Moreover, the YOLOv5 object detection algorithm has several advantages over traditional object detection algorithms such as Fast R-CNN, Faster R-CNN, Mask R-CNN, and SSD. Firstly, YOLOv5 exhibits high real-time performance, enabling real-time object detection in a short period of time for images or videos. Secondly, YOLOv5 employs a simple and easy-to-implement network structure that is relatively lightweight, requiring fewer memory and computational resources. It is suitable for embedded devices and edge computing platforms, reducing the requirements for computational resources and storage space, and improving the efficiency and scalability of the entire system. Additionally, in terms of object detection accuracy, YOLOv5 introduces multi-scale detection techniques, allowing it to handle objects of different scales and enhancing the algorithm’s robustness.

These advantages are crucial in fault detection in power distribution networks, as they ensuring the timely detection and handling of faults, thereby avoiding further damage to the power system. Therefore, in this paper, the focus is on improving the YOLOv5 algorithm to meet the inspection requirements of power distribution networks. The proposed enhancement results in a lightweight and high-precision YOLOv5 network, referred to as Comprehensive-YOLOv5.

The network architecture of YOLOv5 comprises four components: Input, Backbone, Neck, and Prediction. The Input module employs Mosaic data augmentation and adaptive anchor box computation. Mosaic data augmentation combines images by random scaling, cropping, and arrangement to enhance the detection performance for small objects. Adaptive anchor box computation calculates anchor box values based on optimal anchors specific to different training sets. This reduces the disparity between the ground truth boxes and anchor boxes, enhancing the speed of object detection during backpropagation updates.

The Backbone module employs Cross Stage Partial Darknet (CSPDarknet) to extract rich information features from input images. Cross Stage Partial Network (CSPNet) addresses the problem of redundant gradient information in optimizing the backbone of deep convolutional neural network models. By integrating gradient changes into feature maps, CSPNet reduces the model’s parameter size, floating-point operations per second (FLOPS), and overall model size. This optimization leads to improved inference speed and accuracy while reducing the model’s footprint. These design and optimization measures make YOLOv5 a robust and efficient network model suitable for fault detection in power distribution networks.

In YOLOv5, the CSPNet structure incorporates two types of Cross Stage Partial (CSP) structures. The first one, known as Cross Stage Partial 1_X (CSP1_X), is utilized in the backbone network and adopts a residual structure to enhance the learning capacity of the convolutional neural network. The second one, called Cross Stage Partial 2_X (CSP2_X), is used in the neck network and does not employ the residual structure. In the backbone network, the input feature map is processed through the Focus structure. The Focus structure integrates the width (w) and height (h) information into the channel (c) dimension through slicing operations. More specifically, it reduces the width and height by half while increasing the number of channels by four times.

The CSPNet structure introduces the Leaky Rectified Linear Unit (LeakyReLU) activation function, which is an improved version of Rectified Linear Unit (ReLU). During training, when the input value is less than 0, the output after activation remains non-zero. This ensures the activation of some neurons and prevents the problem of parameters never being updated. The LeakyReLU activation function introduces a parameter “a” that controls the slope of negative values. The expression for LeakyReLU function is as follows:

(1) LeakyReLUa(x)=max(ax,x)

In the equation, x represents the input value, and the parameter a is typically set to 0.01.

**Figure 2:**
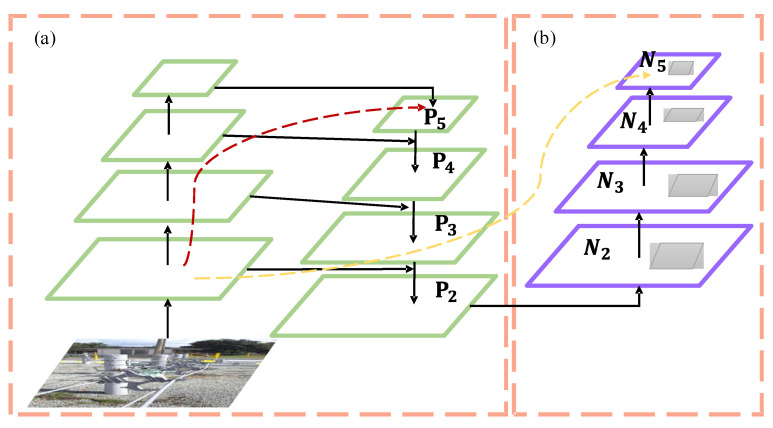
FPN + PANet structure. (**a**) FPN backbone; (**b**) PANet backbone.

The neck network in YOLOv5 incorporates the FPN + PANet structure, as illustrated in Figure 2. In the figure, (a) represents the Feature Pyramid Network (FPN) backbone, while (b) represents the PANet backbone. FPN is primarily utilized to enhance object detection by merging high-level and low-level features, leading to improved detection performance, particularly for small objects.

PANet builds upon FPN by introducing the Bottom-up Path Augmentation structure, which fully utilizes the shallow features of the network for segmentation. This allows the top-level feature maps to benefit from rich positional information derived from the bottom-level features. This improvement enhances the detection performance for larger objects, enabling the model to better recognize objects of varying sizes and scales.

The loss function in YOLOv5 consists of three components: the bounding box regression loss (Loss(coord)), the confidence prediction loss (Loss(conf)), and the class prediction loss (Loss(cls)). The bounding box regression loss is calculated using the GIoU loss function, which quantifies the discrepancy between predicted and ground truth bounding boxes. The confidence prediction loss is computed using the Binary Cross Entropy with Logits (BECLogits) loss function, which evaluates the loss for the probability of object presence. The class prediction loss is calculated using the cross-entropy loss function (BCEclsloss), which measures the loss for class predictions. The specific formulas for these losses are as follows:

(2) $$\mathrm{Loss}={\mathrm{Loss}}_{\left(\mathrm{coord}\right)}+{\mathrm{Loss}}_{\left(\mathrm{conf}\right)}+{\mathrm{Loss}}_{\left(\mathrm{cls}\right)}$$

(3) $${\mathrm{Loss}}_{(\mathrm{coord})\;}=1-\mathrm{GIoU}$$

(4) $$GIoU=IoU-\frac{\left|C/(A\cup B)\right|}{\left|C\right|}$$

(5) $$\begin{array}{cc} {\mathrm{Loss}}_{\left(\mathrm{conf}\right)} & =\sum_{i=0}^{S\times S}\sum_{j=0}^{B}I_{ij}^{obj}\left[\overset{\Lambda }{C_{i}}\log\left(C_{i}\right)+\left(1-\overset{\Lambda }{C_{i}}\right)\log\left(1-C_{i}\right)\right]\\ & -\sum_{I=0}^{S\times S}\sum_{j=0}^{B}I_{ij}^{nobj}\left[\overset{\Lambda }{C_{i}}\log\left(C_{i}\right)+\left(1-\overset{\Lambda }{C_{i}}\right)\log\left(1-C_{i}\right)\right] \end{array}$$

(6) $${\mathrm{Loss}}_{\left(cls\right)}=\sum_{t=0}^{S\times S}\sum_{j=0}^{B}I_{ij}^{\mathrm{obj}\;}\sum_{c\in \mathrm{classes}\;}\left[{\hat{p}}_{i}(c)\log\left(p_{i}\right)\right)+(1-{\hat{p}}_{i}(c)\log(1-p_{i}(c))]$$

In the equation, *S* represents the size of the network. *i* represents the *i*-th grid cell in the feature map, while *j* represents the *j*-th predicted bounding box associated with that grid cell. Subscripts “*obj*” and “*nobj*” denote whether an object exists in the *i*-th grid cell. $C_{i}$ represents the predicted class for the bounding box, while $\overset{\Lambda }{C_{i}}$ represents the true class for the ground truth box. $p_{i}$ represents the predicted object confidence, and ${\hat{p}}_{i}$ represents the actual object confidence.

This paper aims to improve the network structure and loss functions of the original YOLOv5 model to improve the model’s detection accuracy and speed.

### 2.2. GhostNet Convolutional Network

In mainstream deep neural networks, feature maps extracted from input data often contain abundant and occasionally redundant information to ensure a comprehensive understanding of the input data. GhostNet introduces a novel convolutional method known as Ghost convolution, which aims to replace traditional standard convolutions. In comparison to conventional neural networks, Ghost convolution can extract redundant information from feature maps with lower computational cost and higher efficiency.

**Figure 3:**
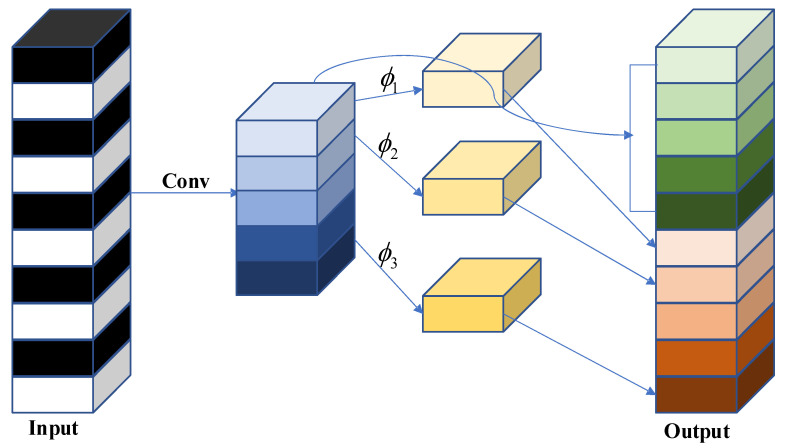
GhostConv module.

Ghost convolution begins by applying a regular 1 × 1 convolution to reduce the dimensionality of the input feature map, resulting in a feature map (referred to as feature map 1) containing redundant information. Subsequently, feature map 1 undergoes an identity mapping and inexpensive non-linear operations Φ. These operations include convolutional layers, batch normalization layers, and ReLU layers. The convolutional layers used in this process are depth-wise separable convolutions, such as 3 × 3 or 5 × 5 convolutions, applied separately to each feature map. Finally, the mapped and non-linear transformed feature maps are concatenated to obtain the complete feature map. The specific schematic diagram of the Ghost convolution module is shown in Figure 3.

Assuming the input feature map size is h⋅w⋅n, the number of linear operations is $h^{\prime }\cdot w^{\prime }\cdot n$, and the final output feature map size is s, with a convolution kernel size of k⋅k. Considering that a Ghost convolution includes one mapping operation, the actual number of linear operations is s−1. Therefore, the number of parameters in a Ghost convolution is determined by the following formula:

(7) $$p_{1}=\frac{n}{s}\cdot c\cdot k\cdot k+(s-1)\cdot \frac{n}{s}\cdot d\cdot d$$

The number of parameters in a regular convolution can be calculated as follows:

(8) $$p_{2}=n\cdot c\cdot k\cdot k$$

The ratio of parameter counts is calculated as follows:

(9) $$r_{c}=\frac{n\cdot c\cdot k\cdot k}{\frac{n}{s}\cdot c\cdot k\cdot k+(s-1)\cdot \frac{n}{s}\cdot d\cdot d}\approx \frac{s\cdot c}{s+c-1}\approx s$$

**Figure 4:**
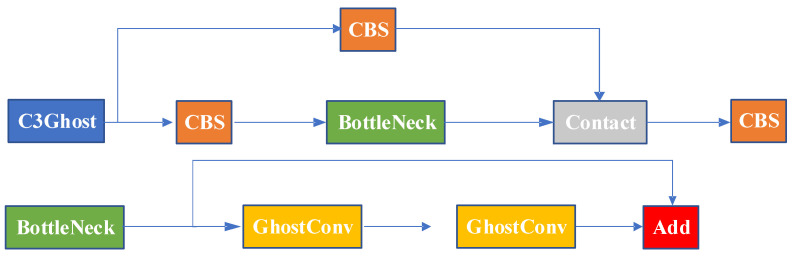
C3Ghost module.

Based on the above discussion, Ghost convolution has a significant advantage over regular convolution in terms of parameter reduction, allowing for model compression and feature extraction at a smaller cost. Building upon Ghost convolution, we designed the C3Ghost structure, which is illustrated in the diagram shown in Figure 4.

In order to address the potential increase in parameter count and computational complexity resulting from subsequent improvements, this paper leverages GhostNet for the reconstruction of the backbone and neck of YOLOv5. The effectiveness of this approach through is validated subsequent experiments. The specific reconstruction operations involve replacing regular convolutions in the backbone and neck with Ghost convolutions and replacing the C3_2 module in the backbone and neck with the C3Ghost module. These reconstruction operations aid in reducing parameter count and computational complexity, thereby enhancing the efficiency and performance of the model.

### 2.3. Bi-Directional Feature Pyramid Network

**Figure 5:**
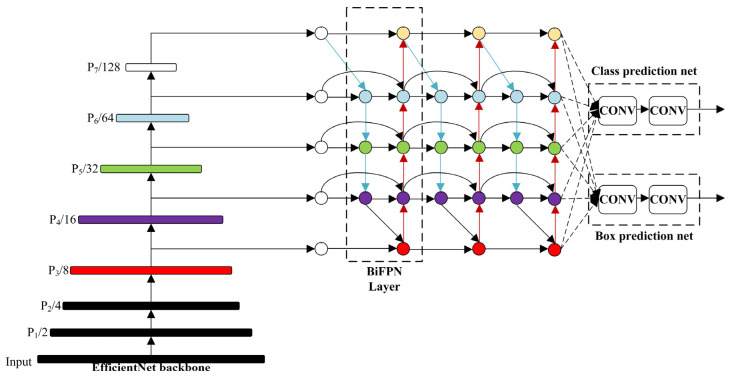
Schematic diagram of the BiFPN structure.

Convolutional neural networks (CNNs) extract target features in a hierarchical manner, with shallow layers capture the spatial information of images and deeper layers containing higher-level semantic information. This hierarchical structure enables CNNs to gradually extract rich feature representations, leading to a more accurate understanding and recognition of targets. However, relying solely on feature extraction at a single resolution can lead to information loss or insufficiency. The purpose of multi-scale feature fusion is to aggregate features from different resolutions, thereby effectively utilizing both shallow and deep information in the network. By fusing multi-scale features, the network can obtain more comprehensive and rich feature representations, leading to enhanced accuracy in object detection and localization, particularly in complex scenes. There are various approaches to achieve multi-scale feature fusion, such as utilizing Feature Pyramid Network, bottom-up path aggregation, bi-directional propagation, and others. These methods introduce appropriate connections and operations in the network, enabling effective communication and fusion of features from different levels. Through multi-scale feature fusion, the network can fully leverage feature information at different scales, thereby enhancing its detection capability for objects of varying sizes and complexities. This leads to improved robustness and generalization performance of the network. Multi-scale feature fusion plays a crucial role in CNNs as it combines the advantages of both shallow and deep features, resulting in enhanced object detection performance. With a well-designed feature fusion method, the network can better comprehend the spatial and semantic information of images, leading to more accurate object localization and recognition. In this paper, we adopt the YOLOv5 algorithm and introduce an efficient and fast feature fusion structure called Bi-directional Feature Pyramid Network (BiFPN), as depicted in Figure 5, to further enhance the inference performance of the model.

Due to the use of Ghost convolution to reduce parameters and computations, there is a trade-off with a decrease in detection accuracy. In order to address this, we have chosen to introduce BiFPN into the Neck network of YOLOv5, replacing the original Path Aggregation Network (PANet). We have constructed the Concat operation to replace the regular Concat layer, thereby enhancing the network’s feature fusion capability through learnable weights.

**Figure 6:**
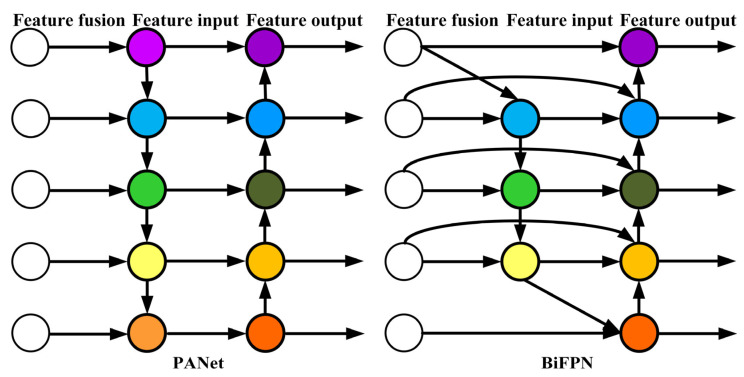
Comparison between PANet and BiFPN structures.

BiFPN is a weighted bidirectional feature pyramid network proposed by Google Brain. Unlike the feature pyramid network (Feature Pyramid Network) that passes features through a single top-down path, BiFPN incorporates a reverse path to convey positional information that might have been lost. It builds upon PANet by removing nodes with only one input, optimizing the network while preserving important information. Additionally, if the input and output nodes are at the same level, an additional edge is added to fuse more features. The comparison between BiFPN and PANet structures is illustrated in Figure 6.

Importantly, BiFPN proposes a weighted feature fusion mechanism that assigns a learnable weight to each path. These weights are continuously updated through feature learning to prioritize more important information. Due to the introduction of bidirectional information flow and adaptive feature weighting, BiFPN may be more complex compared to PANet. This increased complexity may require additional computational resources and time for feature fusion, potentially impacting the training and inference time of the model.

However, the multi-level feature fusion and adaptive feature selection capability of BiFPN can improve the accuracy and robustness of the detection model, effectively eliminating the side effects of accuracy and robustness reduction caused by lightweight model optimization. In this study, we aim to maintain high detection accuracy while improving detection speed. Therefore, we adopt the BiFPN structure to mitigate the accuracy degradation caused by using Ghost convolution to improve inference speed.

### 2.4. Focal Extended Intersection over Union Loss Function

The original YOLOv5 algorithm utilizes the Generalized Intersection over Union (GIOU) loss function to calculate bounding box regression. The GIoU formula is represented as follows:

(10) $$\left\{ \begin{array}{l} GIOU=IoU-\frac{A^{c}-u}{A^{c}}\\ -1\leq GIOU\leq 1 \end{array}\right.$$

In the equation, $A^{c}$ represents the area of the minimum bounding rectangle of the actual box and the predicted box, while *u* represents the intersection area between the actual box and the predicted box.

To address the aforementioned issues of GIoU, we adopt the Focal Enhanced Intersection over Union (Focal-EIoU) loss function instead of GIoU for bounding box regression in our study. The penalty term of Enhanced Intersection over Union (EIoU) separates the influence of aspect ratio in the penalty term, allowing independent calculation of the lengths and widths of the target box and anchor box.

The Focal-EIoU loss function comprises three components: overlap loss, center distance loss, and width-height loss. The first two components follow the methodology of CIOU, while the width-height loss aims to minimize the difference between the widths and heights of the target box and anchor box, resulting in faster convergence. The formula for the penalty term is as follows:

(11) $$L_{EIOU}=L_{IOU}+L_{dis}+L_{asp}=1-IOU+\frac{\rho^{2}(b,b^{gt})}{C^{2}}+\frac{\rho^{2}(w,w^{gt})}{{\left(C^{w}\right)}^{2}}+\frac{\rho^{2}(h,h^{gt})}{{\left(C^{h}\right)}^{2}}$$

In the equation, $C^{w}$ and $C^{h}$ represent the width and height of the minimum enclosing box that covers the two boxes.

Considering the issue of sample imbalance in bounding box regression, where the number of high-quality anchor boxes with small regression errors is much smaller than that of low-quality samples, the training process can be significantly influenced by the large gradients produced by low-quality samples. To address this, we propose a Focal EIOU Loss by combining Focal Loss with EIOU. From the perspective of gradient adjustment, this approach separates high-quality anchor boxes from low-quality ones. The formula for the penalty term is as follows:

(12) $$L_{\mathrm{Focal}-\mathrm{EIOU}}=IOU^{\gamma }L_{EIOU}$$

In the equation, IOU=|A∩B|/|A∪B|, and γ is a parameter controlling the degree of outlier suppression. The Focal-EIOU loss function in this context differs from the traditional Focal Loss by assigning larger losses to higher Intersection over Union (IoU) values instead of focusing on difficult samples. This effectively gives more weight to better regression targets, leading to improved regression accuracy.

The EIOU loss function takes into account the overlapping area, center point distance, and the differences in length and width of the bounding boxes. It addresses the ambiguous definition of aspect ratio based on CIOU and incorporates Focal Loss to handle the issue of sample imbalance in bounding box regression.

Compared to the original GIOU loss function in YOLOv5, the Focal-EIOU loss function achieves higher accuracy and faster convergence.

### 2.5. Network Model of Comprehensive-YOLOv5

**Figure 7:**
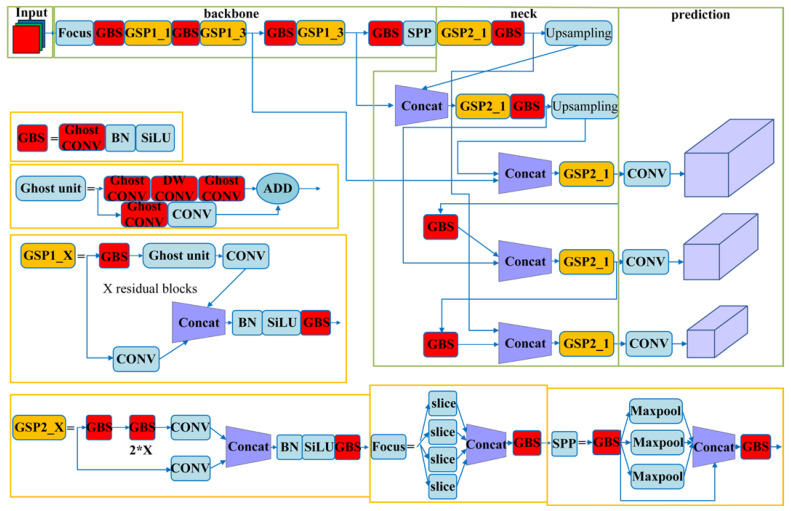
Network Model of Comprehensive-YOLOv5.

Based on the description above, the improved network architecture of Comprehensive-YOLOv5 is shown in Figure 7.

## 3. Experimental Setup and Analysis

### 3.1. Dataset Creation and Anchor Box Selection

#### 3.1.1. Acquisition of Distribution Grid Defect Dataset

In this study, a dataset of 3000 original images of distribution grids was collected in a city. The data collection process involved manual photography and the use of a camera-equipped unmanned aerial vehicle (UAV). The images were captured at a resolution of 608 × 608 pixels and encompassed three types of faults: insulator without loop, cable detachment from insulators, and cable detachment from spacers.

**Figure 8:**
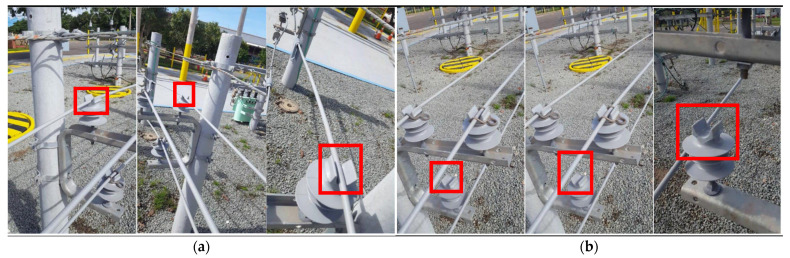

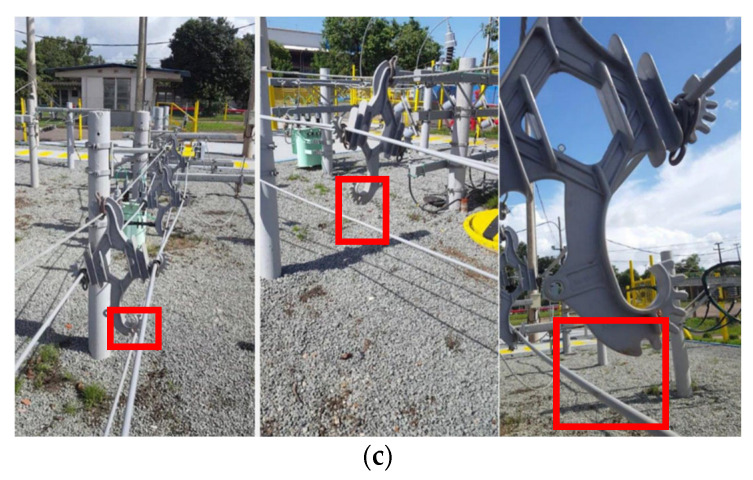
Three Typical Defects in Distribution Grids. (**a**) Insulator Ring Absence; (**b**) Cable Detachment from Insulators; (**c**) Cable Detachment from Spacers.

The faults were photographed from five different angles: left, right, top, bottom, and front, at distances ranging from 20 to 100 cm from the faults. The anchor boxes, which indicate the locations of the faults, were annotated and are depicted in Figure 8.

#### 3.1.2. Data Augmentation and Preprocessing

To mitigate overfitting issues in deep learning models, it is necessary to train the model with a large amount of data samples. During the training phase, the recognition performance of the model can be significantly enhanced by utilizing comprehensive and diverse training data. In order to improve the generalization and anti-interference capabilities of the model, this study conducted various data augmentation and preprocessing operations on the original data before training.

The employed image augmentation techniques include horizontal flipping, vertical flipping, random cropping, random rotation, and color jittering. These operations increase the diversity of the dataset and improve the model’s robustness. After data augmentation, a total of 12,000 images were obtained as the dataset.

Data preprocessing involves a series of processing operations applied to the original data to prepare it for model training. The data preprocessing operations utilized in this study include image scaling, normalization, and contrast enhancement.

Specifically, the images were resized to 416 × 416 pixels to match the input size of the Comprehensive-YOLOv5 model. Additionally, the images were standardized to accelerate model convergence and enhance contrast to improve the visualization of defect areas.

#### 3.1.3. Establishment of Image and Label Database

In this study, version 4.5.9 of the labelImg software was used to annotate the images, generating annotation files in XML format. To convert the annotation files into the corresponding YOLO format, a Python script was developed to process the annotation files. The data was then split into training, validation, and testing sets according to an 8:1:1 ratio.

### 3.2. Experimental Conditions and Training Hyperparameter Settings

The training environment of the target detection algorithm in this experiment is described in Table 1.

**Figure 9:**
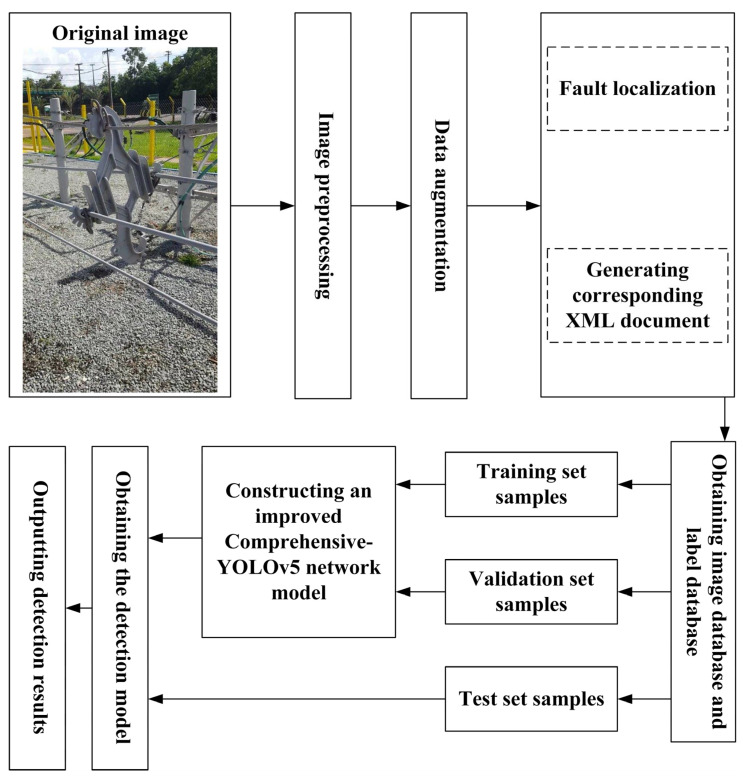
Distribution Grid Defect Detection Process Flowchart.

The Distribution Grid Defect Detection Process Flowchart is shown in Figure 9.

The hyperparameter settings for training the algorithm in this paper are presented in Table 2.

### 3.3. Evaluation Criteria

This paper aims to utilize the Comprehensive-YOLOv5 algorithm as a lightweight model while maintaining high detection accuracy. To assess the algorithm’s speed, frames per second (FPS) and inference time are employed as evaluation metrics. The spatial and temporal complexity of the algorithm is evaluated using floating-point operations (FLOPs) and the size of the model’s weights. The detection accuracy of the algorithm is evaluated using mean average precision (mAP).

FPS represents the number of image frames processed by the algorithm per second and serves as a metric for measuring algorithm performance and efficiency. It reflects the algorithm’s real-time capability and responsiveness. In this paper, the detection time for a single image is calculated using Equation (13), which indirectly utilizes FPS for computation.

(13) $$T_{\mathrm{inference}}=\frac{1}{FPS}$$

mAP is the average value of Average Precision (*AP*) for all classes, used to measure the overall performance of object detection algorithms. mAP is calculated using Equation (14).

(14) $$\begin{array}{l} P=\frac{TP}{TP+FP}\\ R=\frac{TP}{TP+FN}\\ AP=\int_{0}^{1}P(R)dR\\ \mathrm{mAP}=\frac{1}{m}\sum_{i=1}^{m}AP_{i} \end{array}$$

In the equations mentioned above, *R* represents recall, *P* represents precision, and *TP*, *FP*, *FN* represent the quantities of true positive, false positive, and false negative predictions made by the model, respectively.

This paper uses mAP@0.5 and mAP@0.95 as evaluation metrics for detection accuracy, which represent the average precision at IoU thresholds of 0.5 and 0.95, respectively.

In the four mentioned equations, *TP* represents the number of true positive samples predicted correctly by the model, *FP* represents the number of false positive samples predicted incorrectly as positive by the model, *FN* represents the number of false negative samples predicted incorrectly as negative by the model, and m represents the number of label categories.

### 3.4. Morphological Experiment

To validate the effectiveness of each improvement module, four sets of morphological experiments were conducted in this study. The training was performed sequentially, following the experimental environment described in Section 3.2. The best weight files from each training set were selected for experimentation on the validation set. For each experiment, the training was carried out for 300 epochs. The results of the morphological experiments are presented in Table 3.

**Figure 10:**
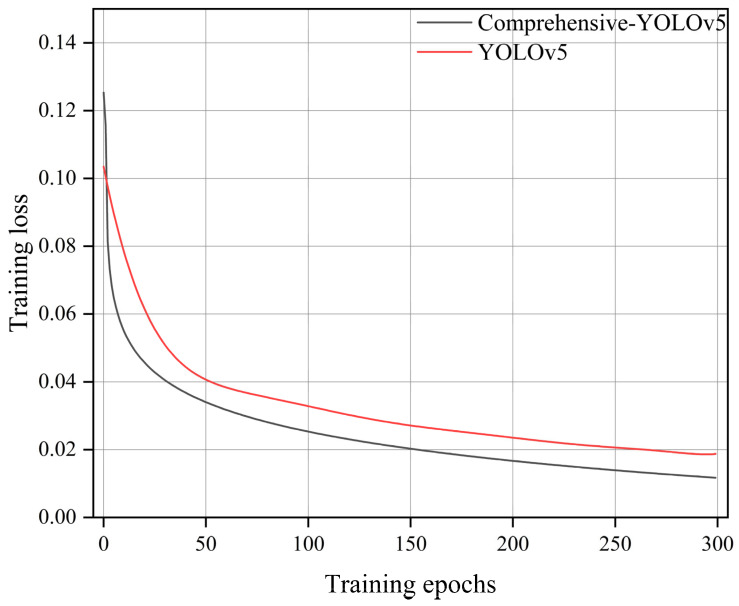
Loss graph.

In the table, “√” indicates the inclusion of a particular module, while “×” indicates the exclusion of that module. Detection time refers to the time required to predict a single image with a batch size set to 1. From Table 1, it can be observed that after incorporating the GhostNet module, the average precision decreased by 1.1 percentage points. However, the FPS increased significantly from 20 to 53, and the model size was greatly reduced. With the addition of BiFPN, although there was a slight decrease in FPS and inference speed, the average precision improved by 2.7 percentage points. After replacing GIOU with Focal-EIOU, the model weight size remained almost unchanged, while there were minor improvements in FPS, inference speed, and average precision. Additionally, as shown in Figure 10, it can be observed that Focal-EIOU optimization of the GIOU loss function significantly improved the convergence speed compared to YOLOv5.

The ablative experiments demonstrate that Comprehensive-YOLOv5, in comparison to the original YOLOv5 model, achieved an overall accuracy improvement of 1.8 percentage points. Furthermore, the FPS increased from 20 to 52, resulting in a significant 160% improvement in inference speed. This improvement in speed makes it feasible to deploy the model on edge devices for fault detection in power distribution networks.

### 3.5. Algorithm Comparison Experiment

To validate the superiority of the proposed Comprehensive-YOLOv5 among similar methods, we trained YOLOv4, YOLOv5, Faster RCNN, DETR, YOLOv5-Lite, and Comprehensive-YOLOv5 using the same hyperparameters on our constructed comprehensive dataset of distribution network line defects. Subsequently, we conducted testing on a computer. The test results are shown in Table 4.

It is evident that Faster RCNN performs worse than other algorithms in various parameters, particularly in terms of long inference time and low frame rate, making it unsuitable for deployment on edge devices. YOLOv4 and YOLOv5 algorithms are among the most popular object detection algorithms, achieving a good balance between accuracy and speed.

The DETR algorithm, utilizing a transformer for self-attention on the backbone feature map, demonstrates a significant improvement over YOLOv5 in terms of performance. However, its detection speed is relatively slower. The YOLOv5-Lite algorithm proposed in literature [36] achieves a detection speed of 62 FPS on edge devices, but at the cost of lower accuracy, indicated by a mAP@0.5 of only 71.1%. This poses challenges in meeting the requirements of precise real-time detection.

In contrast, the proposed Comprehensive-YOLOv5 algorithm, with the highest mAP, achieves a detection speed of 52 FPS, surpassing other detection algorithms. It effectively meets the demands of high-performance and high-accuracy inspection of power transmission lines.

### 3.6. Comparative Analysis of Detection Performance

**Figure 11:**
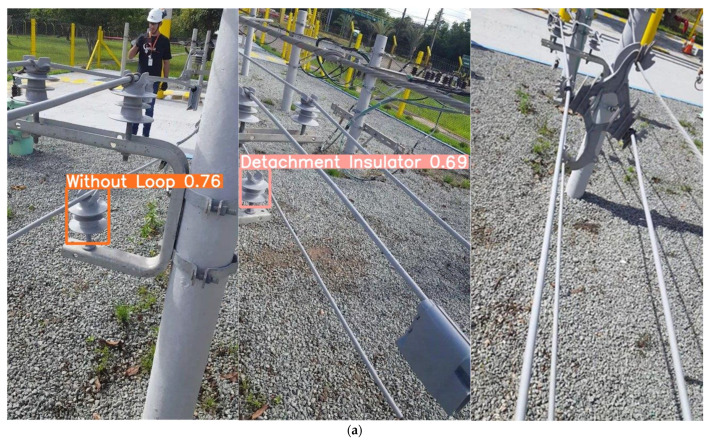

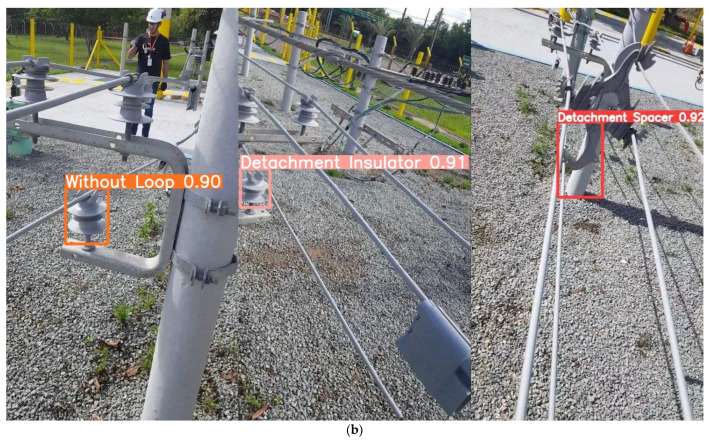
Comparative Detection Results Chart. (**a**) YOLOv5 Detection Results Chart. (**b**) Comprehensive-YOLOv5 Detection Results Chart.

To provide a more intuitive representation of the performance of the improved model, this study selects images of three types of faults from the validation set and compares the detection results between the original and improved models. The comparative analysis results are shown in Figure 11.

From the effectiveness images, it is evident that the Comprehensive-YOLOv5 model demonstrates significantly improved confidence scores compared to the original YOLOv5 model for all types of faults. The confidence scores of YOLOv5 typically range from 0.7 to 0.8, while the Comprehensive-YOLOv5 model achieves confidence scores exceeding 0.9, indicating a substantial improvement in detection confidence.

Furthermore, in the third image depicting the fault of cable detachment from the spacer, where occlusion is present, the YOLOv5 model fails to accurately recognize the fault. However, the Comprehensive-YOLOv5 model successfully detects faults in occluded regions of the power distribution network.

## 4. Conclusions

This study aims to addresses the issues of low detection accuracy and slow speed in existing fault detection models for power distribution networks. To overcome these limitations, we propose an innovative and lightweight approach based on the improved Comprehensive-YOLOv5 model. Our approach is designed to meet the real-time inspection requirements of power distribution network faults while ensuring detection accuracy.

Firstly, the Comprehensive-YOLOv5 lightweight neural network model enables real-time detection of power distribution network faults, specifically identifying three types of faults: “insulator without loop,” “cable detachment from insulator,” and “cable detachment from spacer.” The detection accuracy for these faults reaches 91.4%, 87.1%, and 91.8% respectively. Compared to the original YOLOv5 model, our proposed method significantly reduces the model weight size and achieves a 2.6-fold increase in FPS. The model demonstrates significant improvements in both detection accuracy and speed. Comparative experiments with other models validate the effectiveness of our method.

Furthermore, this method employs Ghost convolution in the backbone and neck, greatly reducing computation time and improving FPS, thus providing a feasible solution for real-time monitoring and edge deployment of power distribution networks.

Lastly, through comparative detection experiments, it is evident that replacing PANet with BiFPN for feature fusion and using Focal-EIOU instead of GIOU in the loss function significantly improves the accuracy, confidence, and detection accuracy under occlusion scenarios of the Comprehensive-YOLOv5 model.

In the context of fault detection in power distribution networks based on Comprehensive-YOLOv5, there are still areas that require further research and effort.

Firstly, there is a need to improve the recognition accuracy of the algorithm while maintaining the current detection speed. Secondly, the algorithm’s recognition capabilities in complex backgrounds, such as rainy, foggy, or low-light conditions, can be enhanced to improve its robustness. Lastly, considering the establishment of a cloud platform to upload real-time detection data of power distribution networks can facilitate better real-time monitoring of fault detection in power distribution networks.

## Figures and Tables

**Table 1:** Algorithm Training Environment.

CPU	GPU	RAM	System Environment
E5 2690v4 ×2 14C 28T 35 MB Cache	RTX 2080 ×2	64GB DDR4 ECC	Ubuntu 20.04 Focal Pytorch1.11 Cuda11.3 + Cudnn8.7.0 Python 3.9.0

**Table 2:** Algorithm Training Hyperparameters.

Hyperparameter	Input Image Size	Epochs	Batch-Size	Learning-Rate	Momentum	Weight Decay Coefficient	Input Channels
Parameter Setting	416 × 416	300	64	0.0001	0.937	0.0005	3

**Table 3:** Results of Comprehensive-YOLOv5 Morphological Experiments.

GhostNet	BiFPN	Focal-EIoU	mAP@0.5/%	mAP@0.95/%	Without Loop	Detachment Insulator	Detachment Spacer	FPS	Inference Speed	Model Weight Size/MB	Parameters	GFLOPs
×	×	×	88.3	45.2	89.4	85.2	90.3	20	5.1	15.5	7,059,201	15.9
√	×	×	87.2	44.8	88.3	82.2	91.1	53	1.9	3.8	2,506,403	5.2
√	√	×	89.9	46.1	91.8	86	91.9	50	2.0	4.0	3,601,412	6.3
√	√	√	90.1	46.9	91.4	87.1	91.8	52	2.1	4.1	3,803,507	6.7

**Table 4:** Comparison of Detection Results among Different Algorithms.

Algorithm	mAP@0.5/%	mAP@0.95/%	Without Loop	Detachment Insulator	Detachment Spacer	FPS	Inference Speed	Model Weight Size/MB	Parameters	GFLOPs
YOLOv4	83.2	43.1	81.4	84.3	83.9	16	6.25	41	21,064,307	43.2
YOLOv5	88.3	45.2	89.4	85.2	90.3	20	5.1	15.5	7,059,201	15.9
DETR	89.1	46.1	90.1	86.6	90.6	8	12.5	159	64,096,782	125.1
Faster RCNN	75.1	40.5	76.5	70.9	77.9	4	25	86	8,942,302	88.1
YOLOv5-Lite	68.1	37.2	70.2	62.8	71.3	40	2.5	2.8	1,536,480	3.6
Cpmprehensive-YOLOv5	90.1	46.9	91.4	87.1	91.8	50	2.0	4.1	3,803,507	6.7

## Data Availability

Not applicable.
